# Does trade liberalization reduce child mortality in low- and middle-income countries? A synthetic control analysis of 36 policy experiments, 1963-2005

**DOI:** 10.1016/j.socscimed.2018.04.001

**Published:** 2018-05

**Authors:** Pepita Barlow

**Affiliations:** Department of Sociology, University of Oxford, Manor Road Building, Manor Road, Oxford, OX1 3UQ, United Kingdom

**Keywords:** Trade liberalization, Child mortality, Developing countries, Sustainable Development Goals, Synthetic control

## Abstract

Scholars have long argued that trade liberalization leads to lower rates of child mortality in developing countries. Yet current scholarship precludes definitive conclusions about the magnitude and direction of this relationship. Here I analyze the impact of trade liberalization on child mortality in 36 low- and middle-income countries, 1963–2005, using the synthetic control method. I test the hypothesis that trade liberalization leads to lower rates of child mortality, examine whether this association varies between countries and over time, and explore the potentially modifying role of democratic politics, historical context, and geographic location on the magnitude and direction of this relationship. My analysis shows that, on average, trade liberalization had no impact on child mortality in low- and middle-income countries between 1963 and 2005 (Average effect (AE): −0.15%; 95% CI: −2.04%–2.18%). Yet the scale, direction and statistical significance of this association varied markedly, ranging from a ∼20% reduction in child mortality in Uruguay to a ∼20% increase in the Philippines compared with synthetic controls. Trade liberalization was also followed by the largest declines in child mortality in democracies (AE 10-years post reform (AE_10_): −3.28%), in Latin America (AE_10_: −4.15%) and in the 1970s (AE_10_: −6.85%). My findings show that trade liberalization can create an opportunity for reducing rates of child mortality, but its effects cannot be guaranteed. Inclusive and pro-growth contextual factors appear to influence whether trade liberalization actually yields beneficial consequences in developing societies.

## Highlights

• Impact of trade liberalization on child mortality is currently unclear.
• Analysis of this relationship using synthetic control method.
• There is no universal association between trade liberalization and child mortality.
• Impact of trade liberalization and child mortality varies substantially.
• Reductions in child mortality greatest in democracies, Latin America, and pre-1990.

## 1 Introduction

Worldwide, rates of child mortality fell by as much as 53% between 1990 and 2015 (You et al., 2015). Despite this progress as many as 5.9 million children under the age of five died in 2015 globally (UNICEF, 2015). A majority of these deaths were attributable to treatable and preventable causes and occurred in low- and middle-income countries (Black et al., 2013; UNICEF, 2015). Thus, reducing child mortality is a key objective in the Sustainable Development Goals (SDGs), adopted by 193 countries in September 2015 (UN, 2015). Scholars have long argued that growth-oriented macro-economic policies can lead to lower child mortality rates (Subramanian et al., 2002; Bettcher and Lee, 2002; Pritchett and Summers, 1996). One such policy is trade liberalization: the removal of restrictions on exports and imports between countries by repealing trade bans or quotas, lowering trade taxes or ‘tariffs’, and eliminating fixed exchange rates (Winters, 2000). Trade liberalization could reduce child mortality through several hypothesized mechanisms, including raising incomes, reducing poverty, and increasing access to medicines and nutritious food (Levine and Rothman, 2006; Bettcher et al., 2000; Blouin et al., 2009). However, trade liberalization could also lead to a rise in child mortality by, for example, increasing the cost of pharmaceuticals and worsening environmental conditions (Blouin et al., 2009). These mechanisms and their impacts on child mortality – for better and for worse – are all supported by varying levels of evidence and, ultimately, whether or not trade liberalization actually leads to a reduction in child mortality is an empirical question.

Yet, two recent reviews published in *Social Science and Medicine* showed that previous studies investigating the relationship between trade liberalization and child mortality were inconclusive (McNamara, 2017; Burns et al., 2016). Prior studies reported contrasting results, used liberalization indicators with weak specificity, and did not adequately address limitations to causal inference when analyzing the impact of trade reforms. Furthermore, prior studies did not examine the scale and potential sources of heterogeneity in the relationship between trade liberalization and child mortality. Here I address these limitations by analyzing the impact of trade liberalization on child mortality in 36 low- and middle-income countries, 1963–2005, using the synthetic control method. I test the hypothesis that trade liberalization leads to lower rates of child mortality, examine the degree of cross-country and temporal heterogeneity, and explore the potentially modifying role of democratic politics, historical context, and geographic location on the magnitude of this relationship.

## 2 Background

### 2.1 Theoretical framework

A large number of studies has identified how trade liberalization could impact on child mortality, for better or for worse, through myriad and complex pathways (Labonté and Schrecker, 2007; Bettcher et al., 2000; Blouin et al., 2009; Barlow et al., 2017b; Bozorgmehr and San Sebastian, 2014). Much like other economic reforms and economic growth (Pritchett and Summers, 1996; Subramanian et al., 2002; Kentikelenis, 2017), trade liberalization can yield effects via changes to health-care and services and via changes to the social, economic and environmental context of a society, which are all important determinants of parental and child well-being (Dahlgren and Whitehead, 1991; Marmot, 2008).

For example, trade liberalization can improve the quality and access to healthcare by facilitating a rise in imports and a reduction in the prices of medical supplies such as vaccines and pharmaceuticals (Bettcher et al., 2000). Trade liberalization may also facilitate the flow of knowledge, technologies, and information that lead to more effective medical treatments and public health programs (Bettcher et al., 2000). Trade liberalization can also lead to higher rates of economic growth and government tax revenue, providing fiscal resources for funding public health-services, thereby expanding access to care and increasing quality (McNeill et al., 2017). These fiscal resources could also be used to supply other public goods and services that are conducive to better health, such as water sanitation and education (Pritchett and Summers, 1996; Caldwell, 2001). Trade liberalization can also raise employment, wages and incomes and reduce poverty which, in turn, increases access to health-sustaining public services (Levine and Rothman, 2006). These changes can also increase access to other goods and services that are essential to sustaining good health, such as nutritious food and housing (Pritchett and Summers, 1996; Subramanian et al., 2002).

Yet conversely, trade liberalization could lead to rising rates of child mortality in low- and middle-income countries. Access to pharmaceuticals and affordability of health-services could decline due to rising pharmaceutical costs arising from the protection of intellectual property rights in international trade agreements (Stiglitz, 2009). Fiscal resources for spending on health-care and other public services could decline if governments are unable to compensate for fiscal shortfalls arising from lower trade tax-receipts by increasing tax revenue from other sources, such as businesses (McNeill et al., 2017; Baunsgaard and Keen, 2010). In addition, trade liberalization can lead to environmental degradation, deteriorating working conditions, greater job insecurity, and more volatile prices (De Vogli, 2011; Blouin et al., 2009). It is also possible that trade reforms lead to widening wage differentials and worsen material conditions, especially among those working in import-competing sectors (Krugman, 2008; Autor et al., 2013), thereby increasing child mortality by increasing inequality and reducing access to health sustaining goods and services among low-income groups (Blouin et al., 2009). Finally, trade liberalization can increase harmful health behaviours such as tobacco and alcohol consumption among parents, thereby reducing children's health and longevity (Friel et al., 2013; Barlow et al., 2017a; Schram et al., 2017).

### 2.2 Effect heterogeneity

Ultimately, the positive and negative effects of trade liberalization may offset one another, leading to no statistically identifiable impact on child mortality. In addition, the impact of trade liberalization on child mortality is likely to take time to accrue due to the time needed for businesses to respond to lower tariffs, co-ordinate and establish production and distribution networks, and expand production (Krugman, 2008). Thus, the effect on child mortality may vary in the post-liberalization era and could only be apparent 5 or 10 years after reforms are implemented.

The impact of trade liberalization is also likely to vary between countries, and socio-political, geographic, and historical factors could influence the magnitude and direction of this relationship. Winters and Martuscelli (2014) showed that trade was correlated with the highest income gains and lowest poverty rates in democracies. Democracies that undergo trade liberalization may also experience greater reductions in child mortality as they experience greater trade and income growth (Besley and Kudamatsu, 2006; Muntaner et al., 2011). Democracies may also ensure that the economic benefits of trade liberalization translate into inclusive public policies that benefit vulnerable groups (Pieters et al., 2016).

In addition, Billmeier and Nanicini reported that liberalizing the economy had a positive effect on economic growth in most low- and middle-income countries, but more recent liberalizations in the 1990s and in Africa had no significant impact (Billmeier and Nannicini, 2013). They suggest that later liberalizers and African economies may have faced greater competition for exporting labour-intensive goods, such as agricultural products or textiles, and lacked growth-enhancing institutions. Thus, trade liberalization may have also lead to greater reductions in child mortality before the 1990s and outside Africa where income gains – and the health benefits that flow from it – were greatest.

### 2.3 Previous literature

A small number of studies have investigated the association between trade liberalization and rates of under-5 and neo-natal mortality. Levine and Rothman (2006) analyzed the association between trade volumes (imports and exports) as a proportion of Gross Domestic Product (GDP) and infant and child mortality rates in 1990 (Levine and Rothman, 2006). The authors found that a 15-percentage point increase in trade as a share of GDP corresponded to approximately 4 fewer child deaths before age 5 per 1000 live births. However, Levine and Rothman did not disaggregate their analysis into different income groups so it is unclear whether their results hold in low- and middle-income countries which often lacked the institutions that translate trade liberalization into greater trade, economic growth and lower poverty (Rodriguez and Rodrik, 2001; Winters, 2000; Billmeier and Nannicini, 2013). Indeed, Gerring and Thacker (2008) showed that the relationship between trade volumes (as a share of GDP) and infant mortality was negative in high-income countries but was not statistically significant in low- and middle-income countries (Gerring and Thacker, 2008). Yet, these findings contrast with the results from an earlier study by Owen and Wu (2007) who found that the negative association between trade and child mortality was strongest among poorer countries, 1960–1995 (Owen and Wu, 2007). However, this relationship was unstable across model specifications.

Previous studies of trade liberalization and child mortality in low- and middle-income countries therefore paint an unclear picture of this relationship. There are three additional limitations in existing scholarship that could also explain this lack of consensus. First, prior studies quantified the associations between child mortality and trade flows rather than trade liberalizing policies. McNamara argued that analyses of trade flows “conflate liberalization for its presumed outcomes” (McNamara, 2017, p.11). Increases in trade are not an inevitable consequence of trade liberalization in low- and middle-income countries which may lack trade-sustaining institutions (Rodriguez and Rodrik, 2001; Winters, 2000). In addition, trade liberalization is promoted through a range of institutions, agreements and policies (McNamara, 2017). These are, in turn, influenced by wider political forces, including power asymmetries within- and between-countries (Ottersen et al., 2014). Thus, studies of trade liberalization acknowledge the role of wider inequities in shaping well-being, and the impact of trade policy cannot be directly inferred from analyses of trade flows.

Second, prior studies estimated the average effect of trade liberalization on child mortality. They did not examine the degree of cross-country and temporal heterogeneity in this relationship, and the potentially modifying influence of socio-political, geographic and historical factors. Third, as Burns noted, no prior studies “claimed to establish causal associations” (Burns et al., 2016, p.9). Valid causal inference requires specifying an appropriate counterfactual: how child mortality would have changed in a country had it not actually liberalized (Morgan and Winship, 2007). This is challenging here as countries which liberalized often differed from countries that did not. For example, Table 1 shows that countries that were open by 1995 were more likely to be democratic and less likely to be engaged in a civil or international conflict than countries which remained closed to trade.

**Table 1:** Rates of child mortality and country characteristics by liberalization status in 1995.

Variable	Mean and standard deviation		Difference (p-value)
	Liberalized (n = 49)	Not liberalized (n = 23)	
Under 5 mortality rate	45.9 (20.2)	55.4 (24.5)	−9.5 (p < 0.10)
Proportion democratic	0.80 (0.41)	0.52 (0.51)	0.28 (p < 0.05)
Proportion in armed conflict	0.00 (0.00)	0.13 (0.34)	0.14 (p < 0.05)

*Notes:* See Table 2 for measurement and data source for each variable.

Inferences based on comparisons between liberalizing and non-liberalizing economies may therefore capture the effect of macro-economic and political differences which can also affect child mortality. Prior studies addressed this issue by estimating fixed-effects regression models that incorporated time-varying observable and time-invariant unobserved differences as controls. However, fixed-effects regressions can lead to inferences that extrapolate beyond what is observed in the data and so are sensitive to modeling assumptions (King and Zeng, 2006). Furthermore, fixed-effects models implicitly assume that the differences between trade liberalizing and non-liberalizing countries can be captured by covariates and country dummies (Acemoglu et al., 2016). But countries that did and did not liberalize could differ in other measurable and un-measurable ways that might, at least partially, account for observed associations.

Here I address these limitations by evaluating the impact of trade liberalization on child mortality in 36 low- and middle-income countries, 1963–2005, using the synthetic control method. I evaluate whether trade liberalization leads to a reduction in child mortality, whether this association varies between countries and over time in the post-reform period, and whether the scale and magnitude of this association is contingent on a country's democratic status, geographic region, and the historical period of trade reforms.

## 3 Methods

### 3.1 Country-level effects

The synthetic control method, developed by Abadie and colleagues, has been used extensively in analyses of social, political, and economic policies, including trade liberalization (Abadie et al., 2010; Billmeier and Nannicini, 2013; Pieters et al., 2016; Rieger et al., 2017; Barlow et al. 2017a, 2018). The synthetic control method is used to estimate the effect of an event or ‘treatment’, like trade liberalization, by approximating a counterfactual from a weighted combination of outcomes in similar countries. To calculate this weighted combination the algorithm identifies the combination of countries that create a counterfactual ‘synthetic control’ unit that resembles the treated country as closely as possible in the pre-treatment period, per Equation (1):

(1) $$\sum_{1}^{k}v_{k}{\left(X_{1k}-X_{0k}W\right)}^{2}$$

Where X_1k_ is the value of variable *k* in the country that liberalized, X_0k_ is a vector containing the values of variable *k* for the un-treated units, and *v*_k_ is a vector of weights that reflects the predictive power of each variable. The algorithm iterates through all possible combinations of country weights, W, and identifies the combination of countries and weights, W*, that minimizes the difference between the value of predictors in the weighted combination of countries ($X_{0k}W$) and in the liberalized ($X_{1k}$) country before the treatment. Variables with higher predictive power on the outcome, captured in *v*_k,_ are assigned greater importance when minimizing this difference.

The effect of trade liberalization is then estimated by calculating the difference between the outcome in the treated country and its synthetic control after the treatment, per Equation (2):

(2) $$\delta_{jt}=\;100\;x\;\left(\frac{Y_{jt}-\;\sum_{c=1}^{C}W^{*}Y_{ct}}{\sum_{c=1}^{C}W^{*}Y_{ct}}\right)$$

Where Y_*jt*_ is the outcome in the treated country *j* at time *t,* and the synthetic control counterfactual, W* Y_*ct*_, is the weighted outcome in comparison units *c* = 1, …,C according to weights W* as identified above. Thus, $\delta_{jt}$ is the percentage difference in child mortality in the liberalizing country compared with the synthetic control.

To estimate the average effect of trade liberalization across all episodes I follow Acemoglu et al. (2016) in estimating each country-level effect and then calculating the mean of these estimates across all trade liberalization episodes. I estimate this mean at 5 and 10 years post-liberalization and across the full post-treatment period. Average effect estimates should contain more information when the synthetic control provides a better approximation to the counterfactual in the liberalizing countries (Acemoglu et al., 2016). Following Acemoglu et al., 2016 I therefore calculated a weighted-average of treatment effects in which I assign higher weights to estimates from models with a lower prediction error (see Appendix 1). I also follow Acemoglu et al. in excluding from this average and subsequent analyses all effect estimates based on models with a ‘high’ prediction error of more than √3 times the average Root Mean Squared Prediction Error (RMSPE) in the pre-treatment period. As my results may be sensitive to this exclusion criterion I also conducted my analysis using two alternative pre-liberalization RMSPE thresholds: i) greater than the average RMSPE, and ii) greater than 3 times the average RMSPE.

### 3.2 P-values and inference

A limitation of the synthetic control method is that standard methods for assessing the significance of country-level and average effect estimates are not suitable because the number of countries in the sample is too small (Abadie et al., 2010). To evaluate significance of the average liberalization effect I follow Acemoglu et al. (2016) in comparing my estimate to a 95% confidence interval (CI) of effects in ‘placebo’ experiments. To construct this CI I first drew a random sample of 20-years of data in 32 countries that did not liberalize; each sample comprised the same number of countries that actually liberalized in my sample and were not excluded due to a high RMSPE. I estimated a ‘placebo’ effect in each country as if it had liberalized in the middle of the 20-year period, and calculate the mean of these effects in the sample. I then repeated this process by sampling with replacement 5000 times. I evaluated the significance of the average liberalization effect by comparing the mean effect from countries that actually liberalized to the distribution of mean effect sizes in the 5000 samples. The average effect of liberalization is ‘significant’ at the 5% level if it does not belong to the interval that contains the [2.5, 97.5] percentiles of the effect of trade liberalization in the 5000 placebo samples.

When examining individual country-level effects I follow Abadie and Gardeazabal (2003), Abadie et al. (2010) in calculating ‘placebo’ effects as above in every country in each pool of comparison countries. I then calculate ‘pseudo *p*-values’: the proportion of placebo effect sizes in a country's pool of comparison countries that are at least as large as the actual effect in the treated country. Larger proportions would undermine my confidence that the observed effect is indeed driven by the treatment rather than unobserved changes in the post-treatment period that also affected other countries.

Finally, I disaggregate the average treatment effects according to whether the liberalizing country was a democracy, its geographic region, and the decade of reform, and perform a series of robustness checks to test the sensitivity of my results to my sample and model specification.

### 3.3 Data sources and measurement

Table 2 summarises the data sources and measures used in my analysis. My measure of child mortality is the number of babies and children per 1000 live births who died before reaching the age of five in a given year. These data are taken from the UN Inter-Agency Group for Child Mortality Estimation (IGME, 2017). A disadvantage of these data is that they are partially based on simulations. These mortality estimates are nevertheless widely used in cross-national analyses and policy evaluations because of their comparability (Rieger et al., 2017; Moreno-Serra and Smith, 2015; Pieters et al., 2016; Wigley, 2017). In addition, Wigley noted that “child mortality often results from causes that are comparatively easier and less costly to prevent or treat (through access to clean water, oral rehydration, antibiotics, ante and post-natal care etc.)” (Wigley, 2017, p. 142). Consequently, child mortality should be responsive to changing economic circumstances following trade liberalization.

**Table 2:** Data sources and measures.

Variable	Measure	Source
Child mortality	Number of new-born babies per 1000 live births who died before age 5	UN Inter-Agency Group for Child Mortality Estimation, 2017
Trade liberalization	A binary indicator of whether a country meets at least one of the following conditions (1 if yes, 0 otherwise): i) average tariffs exceed 40%, ii) nontariff barriers cover more than 40% of its imports, iii) it has a socialist economic system, iv) the black market premium on the exchange rate exceeds 20%, v) many of its exports are controlled by a state monopoly	Wacziarg and Welch, 2008
Economic development	Real gross domestic product per capita, in 2002 US dollars, adjusted for inflation and differences in purchasing power	IMF World Economic Outlook, various years
Democracy	A binary indicator of whether a country is has a score on the Polity2 Index (a measure of democratization that ranges from −10 to 10) of greater than 0 (1 if yes, 0 otherwise)	Polity IV database, Marshall and Jaggers, 2002
Urbanization	The total population living in urban dwellings a proportion of the overall population, as a percentage	World Bank World Development Indicators, 2016
Female education	The proportion of females who have completed the last year of primary school or higher, as a percentage	Barro and Lee, 2017
Population growth	The rate of growth of midyear population from the same date in the previous year, expressed, as a percentage	World Bank World Development Indicators, 2016
Conflict	A binary indicator of whether a country was involved in a conflict with more than 1000 deaths in the given year (1 if yes, 0 otherwise)	Gleditsch et al., 2002

*Notes:* See Bibliography for full references.

To measure trade liberalization I use an indicator originally developed by Sachs and Warner and later updated by Wacziarg and Welch (Sachs et al., 1995; Wacziarg and Welch, 2008). According to this indicator, a country is considered closed to trade in a given year if at least one of the following five conditions that considerably constrain a country's trade is met: average tariffs exceed 40%, nontariff barriers cover more than 40% of its imports, it has a socialist economic system, the black market premium on the exchange rate exceeds 20%, and a majority of its exports are controlled by a state monopoly. Thus, my dicohotomous liberalization indicator captures a policy change or changes that reduce these constraints on international trade.

I selected predictor variables for matching countries and estimating the synthetic control based on previous studies of child mortality. These variables included a measure of economic development, Gross Domestic Product (GDP) per capita, which can impact child mortality by affecting government resources for expenditure on health and other public services that affect health in low-income countries, like sanitation facilities or education (Subramanian et al., 2002). It can also capture poverty levels, incomes and access to goods and services that sustain child health, such as nutritious food or housing (Omran, 1982; Pritchett and Summers, 1996). In addition, I include a binary measure of whether a country is democratic or not, as democratic regimes can have public policies that are especially beneficial to vulnerable groups (Pieters et al., 2016). I incorporate a measure of urbanization, as access to public goods and health infrastructure is more difficult in rural areas, and of female education, which can impact child health through, for example, increased health care utilization and increased knowledge about disease-preventing behaviours (Aizer and Currie, 2014; Caldwell, 1979; Black et al., 2007). Finally, I follow Pieters et al. (2016) in incorporating a measure of population growth, which can strain public and health services, and of the presence of armed conflict in a country, which can affect mortality directly through physical violence and indirectly by reducing incomes and access to essential infrastructures (Gleditsch et al., 2002).

### 3.4 Sample specification

To construct the analytic sample I first identified all countries that liberalized since 1960 (when data were first available) and had available data 10 years before and after liberalization. Next, I restricted the sample to liberalization episodes where data were also available in at least 2 comparison countries that remained closed throughout the same 20-year period. After applying these criteria my analytic sample comprised 36 trade liberalization episodes (see Appendix 2). The study period begins in 1963, 10 years before the first liberalization episode, and ends in 2005, 10 years after the last liberalization episode for which I was able to identify 2 comparison countries with available data.

## 4 Results

### 4.1 Synthetic control analysis

Out of the 36 trade liberalization episodes included in my analytic sample, 32 models had a sufficiently low prediction error and were included in the analyses presented below. As shown in Appendix 3, the weighted synthetic control units more closely resembled treated countries before they liberalized compared with an un-weighted mean of un-treated countries.

**Fig. 1:**
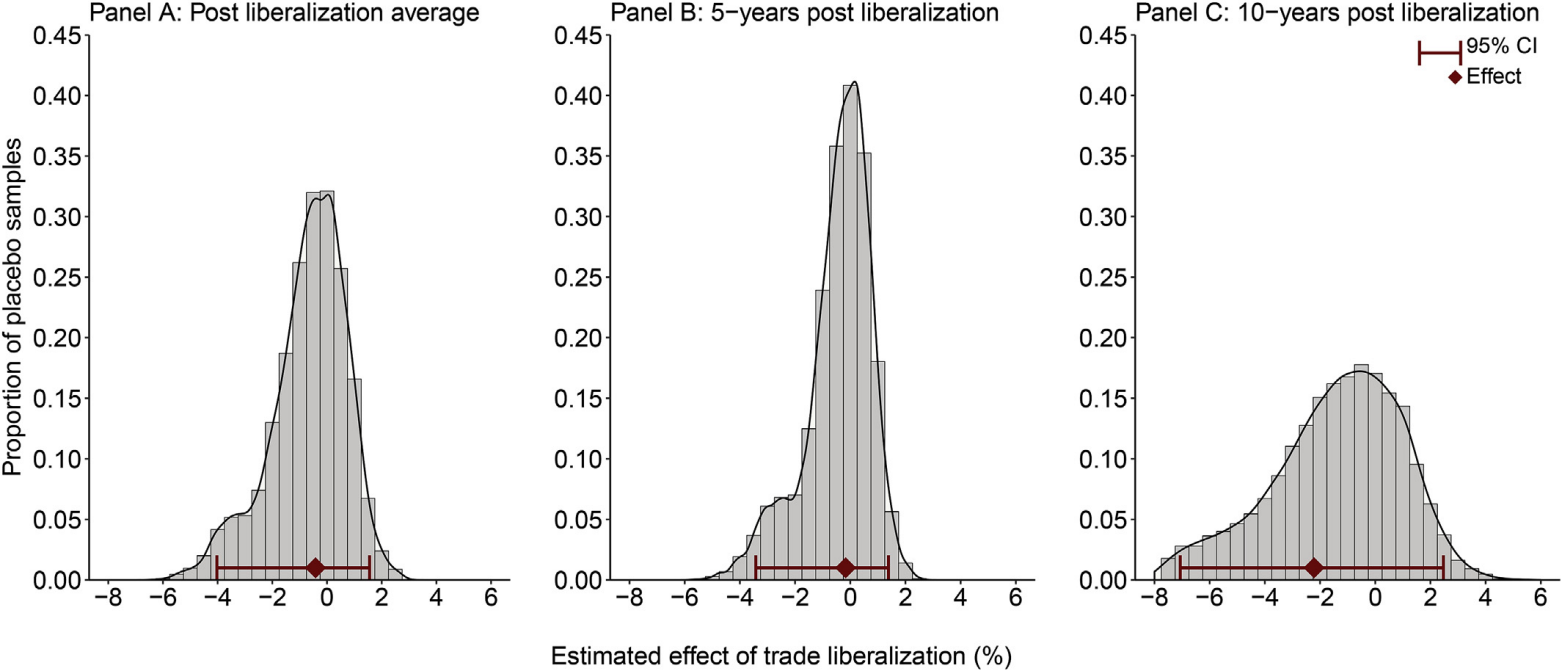
Impact of trade liberalization on child mortality: estimates and 95% confidence intervals for post-reform average effect and 5- and 10-years post-reform effect. *Notes:* 95% confidence intervals are estimated by calculating the mean effect in 5000 placebo samples of 32 ‘fake’ liberalization experiments. Like the average effect estimates, the means of these placebo samples effects were weighted so that weights correspond to each model's prediction error. See Appendix 1 for further detail.

Panels A–C in Fig. 1 show the average effect of trade liberalization on child mortality and the 95% CIs for these estimates. Panel A shows that child mortality was, on average, 0.15% (95% CI: −2.04%–2.18%) lower in countries that liberalized compared with synthetic controls in the post-liberalization period. Panel B shows that this effect had a comparable magnitude 5-years post-reform (Average effect (AE): −0.17%; 95% CI: −3.42 to 1.38). At 10-years post-liberalization the effect was slightly larger: child mortality was on average 2.63% (95% CI: −7.07 to 2.48) lower in countries that liberalized compared with synthetic controls (Panel C). However, all three effect estimates were statistically insignificant: they are within the 95% CI of effect estimates in 5000 samples of ‘placebo’ experiments.

### 4.2 Effect heterogeneity

Fig. 1 obscures substantial cross-country heterogeneity in the estimated effect of trade liberalization. Table 3 shows that the estimated effect of trade liberalization 10-years post-reform ranged by as much as ∼40%, from a 19.5% reduction in child mortality in Uruguay to a 20.8% increase in child mortality in the Philippines. These effects were larger than 5% placebo effects in 12 out of 32 countries.

**Table 3:** Synthetic control results by country.

Country	Average effect (%)	Effect after 5 years (%)	Effect after 10 years (%)	RMSPE	Pseudo p-value
Uruguay	−11.09	−10.69	−19.53	0.18	0.16
Brazil	−8.25	−7.95	−15.56	0.16	0.05
El Salvador	−6.10	−5.13	−14.60	0.01	0.00
Bolivia	−6.19	−6.48	−8.73	0.68	0.38
Peru	−2.94	−2.74	−6.64	0.23	0.32
Ghana	−3.18	−3.07	−6.29	0.07	0.03
Mexico	−4.57	−5.14	−6.21	0.29	0.33
Costa Rica	−1.93	−0.97	−5.37	0.13	0.08
Albania	−2.34	−2.23	−5.22	0.03	0.03
Paraguay	−1.94	−1.77	−4.77	0.04	0.00
Mauritania	−2.40	−2.20	−4.71	0.37	0.35
Jamaica	−1.69	−1.61	−3.99	0.11	0.05
South Africa	−1.43	−1.47	−1.88	0.11	0.11
Honduras	−0.57	−0.53	−1.27	0.01	0.00
Kenya	−0.17	−0.13	−0.68	0.20	0.76
Guatemala	−0.19	−0.13	−0.67	0.11	0.65
Ecuador	0.11	0.22	−0.54	0.07	0.59
Cameroon	−0.26	−0.40	−0.30	0.10	0.46
Mali	0.53	0.56	0.53	0.03	0.03
Benin	0.12	0.04	0.96	0.03	0.08
Guyana	1.51	1.57	1.63	0.61	0.51
Nicaragua	0.85	0.82	1.96	0.02	0.03
Poland	1.85	1.89	3.04	0.04	0.05
Uganda	1.13	0.89	3.20	0.10	0.03
Niger	2.13	2.19	3.27	0.23	0.16
Mozambique	0.22	−0.19	3.93	1.69	0.84
Zambia	4.09	4.77	4.14	0.28	0.06
Colombia	2.13	2.21	4.44	0.01	0.00
Bulgaria	2.26	2.20	4.95	0.05	0.03
Hungary	4.70	4.66	9.15	0.03	0.00
Argentina	7.11	6.58	14.71	0.02	0.06
Philippines	8.51	7.36	20.81	0.24	0.00

*Notes:* Pseudo p-values show the proportion of placebo effects in a country's pool of comparison countries that are at least as large as the actual effect in the treated country.

**Fig. 2:**
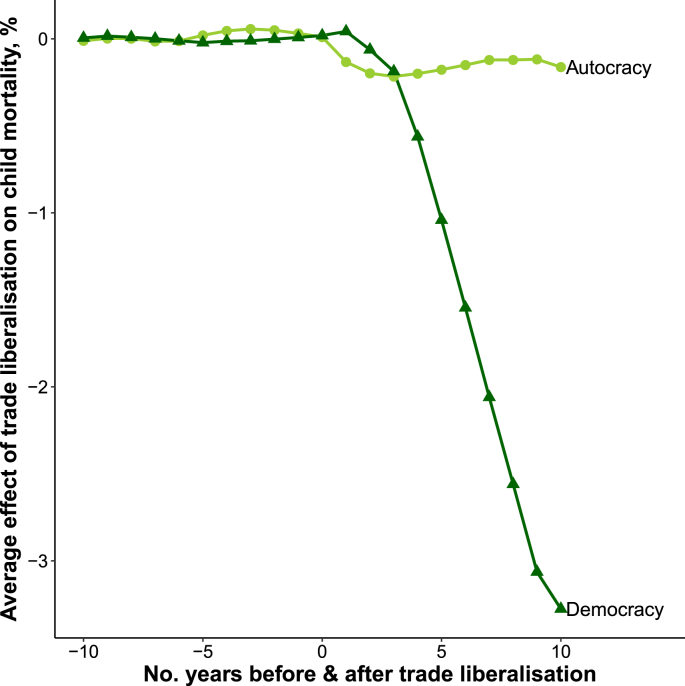
Effect of trade liberalization on child mortality by democratic status. *Notes:* Figure shows normalized average effects where the difference between child mortality in treated countries and synthetic controls was first normalized so that the year of liberalization = 1. These estimates were then averaged using the same RMSPE-weighting procedure as my main analysis (see Appendix 1).

**Fig. 3:**
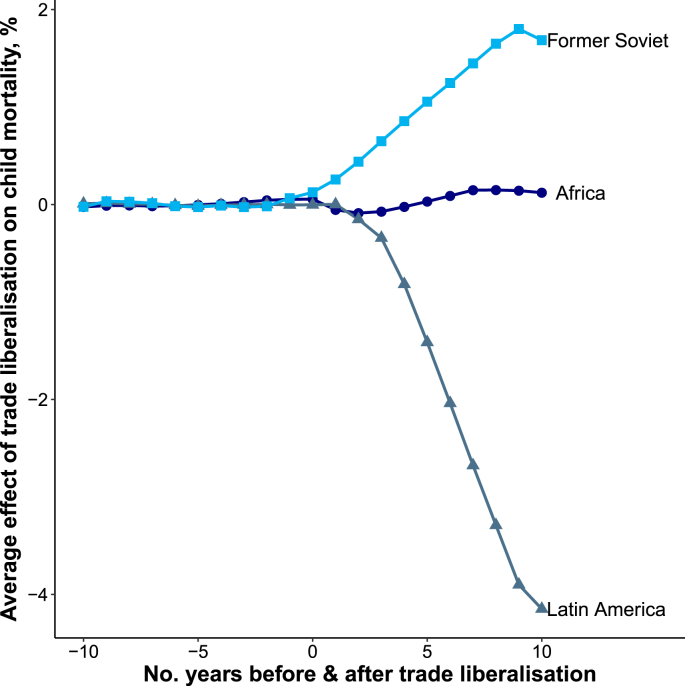
Effect of trade liberalization on child mortality by region. *Notes:* Figure shows normalized average effects where the difference between child mortality in treated countries and synthetic controls was first normalized so that the year of liberalization = 1. These estimates were then averaged using the same RMSPE-weighting procedure as my main analysis (see Appendix 1).

**Fig. 4:**
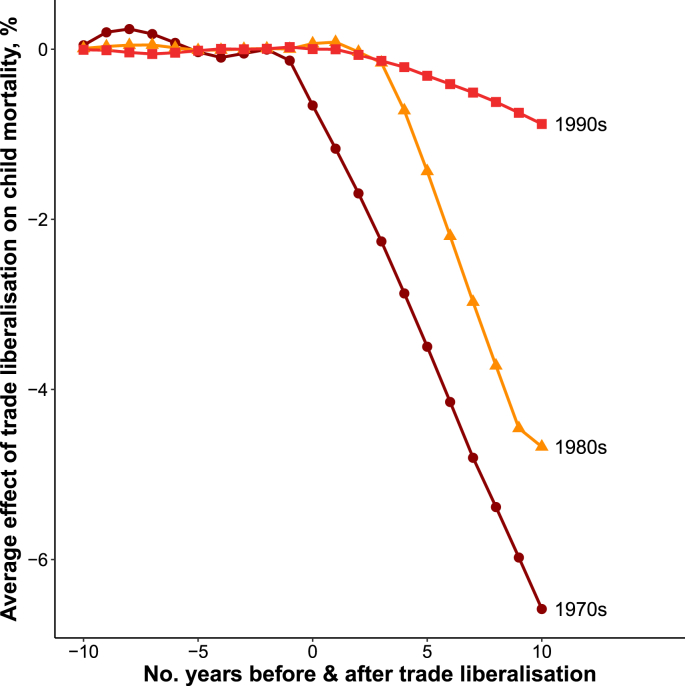
Effect of trade liberalization on child mortality by decade. *Notes:* Figure shows normalized average effects where the difference between child mortality in treated countries and synthetic controls was first normalized so that the year of liberalization = 1. These estimates were then averaged using the same RMSPE-weighting procedure as my main analysis (see Appendix 1).

There were marked differences in the estimated effect of trade liberalization according to a country's democratic status, region, and year of liberalization. Fig. 2 shows that trade liberalization was associated with a decline in child mortality in democracies (Average effect 10-years post reform (AE_10_): −3.28%) whereas there was almost no change in child mortality in autocracies (AE_10_: −0.17%). Fig. 3 shows that trade liberalization was followed by substantial declines in child mortality in Latin America (AE_10_: −4.15%), a slight rise in child mortality in Former Soviet countries (AE_10_: 1.68%), and no appreciable change in child mortality in Africa (AE_10_: 0.12%). Fig. 4 shows that trade liberalization was followed be the largest declines in child mortality in the 1970s (AE_10_: −6.58%) and slightly smaller declines in the 1980s (AE_10_: −4.67%). Trade liberalization episodes in the 1990s were followed by a comparatively modest fall in child mortality (AE_10_: −0.87%).

### 4.3 Robustness checks

I conducted a series of robustness checks to assess the sensitivity of my results to my sample and model specification. Appendix 4 shows the average effect of trade liberalization using two alternative thresholds for excluding cases with high prediction error: greater than the average RMSPE, and greater than 3 times the average RMSPE. Appendix 4 shows that my results were consistent across alternative exclusion criteria: the average effect of trade liberalization was between 0.0% and −1.0% and remained well within the 95% CI.

I originally estimated the average impact of trade liberalization on child mortality by assigning weights to each country according to the model's pre-treatment prediction error. The average effect estimates could therefore be driven by a small number of countries with exceptionally good model fit and very high weights. To test whether this affected my findings I re-estimated my results giving all countries equal weight. This reduced the estimated average effect of trade liberalization on child mortality from −0.15% to −0.59% as the impact of trade liberalization on child mortality was large and negative in a small number of countries with higher prediction error. Nevertheless, the results were consistent with my main findings: the estimated effect was slightly below zero and within the 95% CI of placebo effects in all post-reform time periods (see Appendix 4-5).

**Fig. 5:**
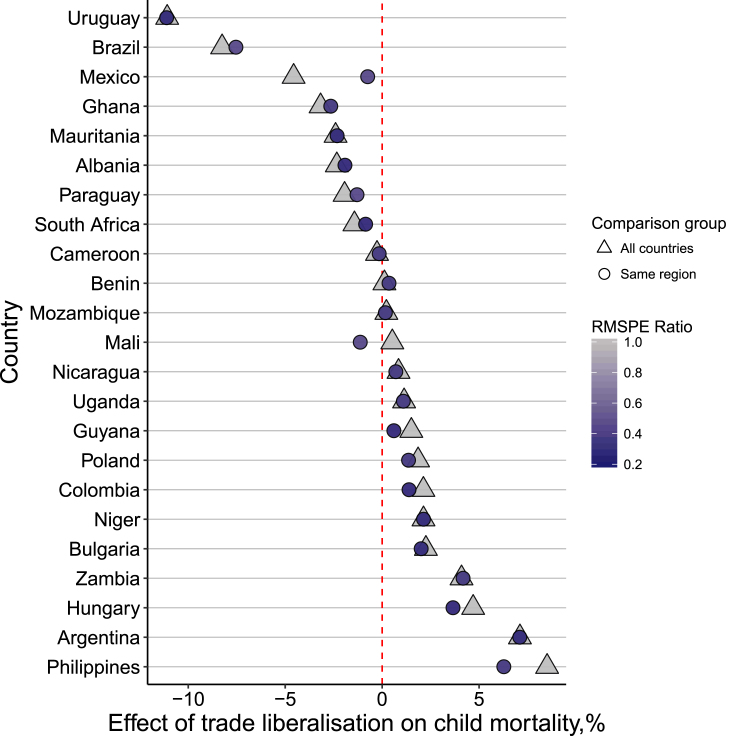
Intra-regional comparisons. *Notes:* The RMSPE Ratio is the ratio of the pre-intervention RMSPE in the model using the specified comparison group to the RMSPE in my original specification. Higher ratios (lighter blue to light grey) indicate better model fit. (For interpretation of the references to colour in this figure legend, the reader is referred to the Web version of this article.)

The synthetic control algorithm assigns weights to countries in a donor-pool comprising all other low- and middle-income countries with available data that did not liberalize. However, comparison countries may differ from the treated country with respect to factors related geography and possibly culture, which could undermine the validity of this comparison. Following Billmeier and Nanicini (2013) I evaluated whether this affected my results by restricting each treated country's donor-pool of comparison countries to those within the same geographic region as the treated country. As shown in Fig. 5, applying this restriction has a cost: the RMSPE of these models was high relative to the original sample specification. However, the direction of the estimated effect of trade liberalization on child mortality was similar in all cases except Mali. Excluding the estimated effect for Mali from the average effect estimations did not substantively alter my estimate of the average effect of trade liberalization, which remained close to zero and within the 95% CI (AE = 0.62%, AE_5_ = 0.72%, AE_10_ = 0.83%).

Finally, the estimated impact of trade liberalization in each country could be attributable to unobserved changes in an un-treated country or countries that were assigned high weights in the synthetic control. To test this possibility I performed a ‘leave-one-out’ analysis in which I iteratively re-estimated the synthetic control results in each country. In each iteration I omitted one un-treated country from the pool of comparison countries and then re-estimated each liberalization effect. Appendices 6-7 plot the results from this analysis. In most countries the effect of trade liberalization on child mortality differed across sample specifications. However, the leave-one-out iterations that produced the largest deviations from my main results had very high prediction error relative to my original models, making these results less valid. In contrast, effect estimates had the same sign, significance, and a similar magnitude to my main results in alternative donor-pool specifications with a pre-treatment prediction error that was as low as my main analysis.

## 5 Discussion

### 5.1 Summary

This analysis has produced three important findings. First, there was no universal association between trade liberalization and child mortality in low- and middle-income countries between 1963 and 2005. Second, the magnitude, direction, and significance of the relationship between trade liberalization and child mortality varied substantially from country to country, ranging by as much as ∼40% across all liberalization episodes. Third, trade liberalization was associated with the largest declines in child mortality in democracies, in Latin America, and in the 1970s and 1980s. Effect sizes were modest in autocracies, in Africa, and in countries which liberalized in the 1990s.

This study advances the long-standing debate about the impact of trade liberalization on child mortality in low- and middle-income countries in several ways. First, I analyzed the impact of trade policy rather than trade flows and, second, I used quasi-experimental methods that strengthen the quality of evidence that informs this debate. Third, I showed that the magnitude and direction of the impact of trade liberalization on child mortality varied considerably during the post-reform period and from country to country. Fourth, I showed that the broader socio-political, geographic and historical context may influence whether liberalization leads to a reduction in child mortality or not. Taken together, these results show that trade liberalization had no universal association with child mortality, but that inclusive, pro-growth contextual factors appear to influence whether trade liberalization actually yields beneficial consequences.

These findings also have important implications for broader debates about the impacts of trade liberalization on well-being in low- and middle-income countries, especially among vulnerable groups. Child mortality is often treated as proxy for other outcomes, such as overall child health, the well-being of the poorest members of society, and health equity (De Looper and Lafortune, 2009; Yazbeck, 2009; Wigley, 2017). In addition, child health is a crucial determinant of educational outcomes, labour productivity and, consequently, future economic growth (Soares, 2005; Bleakley, 2010; Baird et al., 2016). Thus, my analysis of child mortality also shows, indirectly, how trade liberalization has markedly heterogeneous effects on child health, the wellbeing of the poorest in society, health equity, and the long-run economic growth potential that flows from better health, and that these effects can be most beneficial when trade reforms were implemented in inclusive, pro-growth contexts.

A critical question arising from this study is precisely why did the impact of trade liberalization vary to such a large extent between countries and from decade-to-decade? There are several possible explanations. Countries which liberalized in later decades may have already developed to a point where the returns to child mortality of further economic growth had substantially diminished (Preston, 1975; Pritchett and Summers, 1996), or where other factors were more important for sustaining economic growth (Durlauf et al., 2005). It is also plausible that the requirements imposed on trade liberalizing countries via free trade agreements since the 1990s - such as increased intellectual property right protections (Baldwin, 2011) - limited access to medicines and so offset the benefits of trade reforms (Friel et al. 2014). In addition, post-1990 liberalizers may have faced greater competition for exporting labour-intensive goods, such as agricultural products or textiles, compared with countries who were among the first developing countries to liberalize in earlier decades (Billmeier and Nannicini, 2013).

Finally, liberalization after the 1990s, in autocracies, and outside Latin America may associate with a lack of social and political arrangements and policies that sustain economic activity and translate the economic benefits of liberalization into lower poverty and improved child health. Specifically, this includes policies that reduce barriers to creating new business and helping workers find new and better jobs, investments in infrastructure, safety nets to protect the livelihoods of those who suffer unemployment, and educational reforms that foster skill acquisition, wage growth, and employment (Billmeier and Nannicini, 2013; Winters et al., 2004; Zagha and Nankani, 2005). Future research should investigate the distinctive and potentially interactive role of these factors in ensuring that trade liberalization fosters a reduction in child mortality.

### 5.2 Limitations

This analysis has several limitations. First, quasi-experimental identification is not possible without assumptions. The synthetic control methodology assumes that the causing factor does not affect control observations, the stable unit treatment value assumption (‘SUTVA’). It may be that trade liberalization had an indirect effect on other countries due to trade diversion away from closely competing countries that remained closed. However, Cavallo argued that SUTVA is unlikely to affect synthetic control estimates as controls are composed of several countries (Cavallo et al., 2013), so my estimates do not rely on a comparison with each country's single closest competitor.

Second, trade liberalization is not randomly assigned (Rodriguez and Rodrik, 2001). This could create issues when evaluating the effect of trade liberalization if factors leading to liberalization were also correlated with child mortality. However, the synthetic control methodology can address issues associated with countries ‘selecting into’ trade liberalization because it does not require exogenous assignment to treatment; it only assumes that the precise year of adoption is exogenous (Hope, 2016). This is because the synthetic control units are constructed to match countries as closely as possible on the outcome and, consequently, observed and unobserved factors that affect child mortality in the pre-liberalization period (Hope, 2016). This means that potential sources of selection bias are taken into account when constructing the synthetic control units.

Third, it is possible that one or more major events or policy changes occurred simultaneously or after trade liberalization and so account for my results. Additional single-country case-studies using synthetic control methods may help to address this by enabling researchers to combine a systematic, data-driven algorithm for selecting comparison countries with the high-level of granularity that is necessary for identifying co-inciding policy changes (Abadie et al., 2015).

Fourth, my synthetic control estimates identify only the aggregate impact of trade liberalization on child mortality without investigating the mechanisms of transmission. Fifth, comparative, individual-level data were not available for a sufficient number of years or countries pre- and post-trade liberalization, precluding any analysis of socio-economic disparities. Finally, due to methodological constraints I was only able to estimate the impact of trade liberalization in 32 countries that liberalized before 1995. Furthermore, the synthetic control method assumes that the relationships between predictors and child mortality are the same in the pre- and post-liberalization period. My results may therefore have limited external validity. Future research is necessary to address these limitations by evaluating whether these results hold elsewhere, the specific mechanisms through which trade liberalization leads to observed associations, and the socio-economic groups affected.

### 5.3 Conclusion

In summary, my analysis has shown that trade liberalization can lead to lower rates of child mortality in low- and middle-income countries, but inclusive, pro-growth contextual factors appear to influence whether trade liberalization actually yields these effects. These findings have important implications for policy. The UN SDGs target further trade liberalization in low- and middle-income countries and argue that it can serve as an “engine” (UN, 2015, p.87) for achieving other goals, including reducing child mortality. The results from my analysis suggest that further trade liberalization may indeed create an opportunity for reducing child mortality in low- and middle-income countries. But, its beneficial effects cannot be guaranteed.

## Funding

PB was funded by a Wellcome Trust Society and Ethics Doctoral Studentship (WT108696MA). The funder had no role in study design, data collection and analysis, decision to publish, or preparation of the manuscript.

## Footnotes

Appendix A Supplementary data related to this article can be found at https://doi.org/10.1016/j.socscimed.2018.04.001.
