# Supplementary material for: Does trade liberalization reduce child mortality in low- and middle-income countries? A synthetic control analysis of 36 policy experiments, 1963-2005
Source: Soc Sci Med. 2018 May;205:107–15. doi: 10.1016/j.socscimed.2018.04.001 (PMC5956309; doi:10.1016/j.socscimed.2018.04.001)
Supplement: appendix [file mmc1.docx]

# Appendix

Appendix 1. Effect weighting

When calculating the average effect of trade liberalization on child mortality I follow Acemoglu et al. (2016) in assigning weights to each country-level effect estimate according to the model fit in the pre-treatment period, measured as the Root Mean Squared Prediction Error (RMSPE). Country-level effect estimates based on models with a better fit are assigned greater weights by first taking the reciprocal of the model’s RMSPE in the pre-intervention period per Equation 1:

Equation 1. $\rho_{j}=\frac{1}{\sqrt{\sum_{t=T-10}^{T-1} ({Y_{jt}- \sum_{c=1}^{C} w^{*}Y_{ct})}^{2}}}$

Where *j* refers to the liberalizing countries j=1,...,J, c=1,…,C are their comparison countries, and *t* refers to the year in the pre-treatment period, *t* =T-10,…,T-1 (T=0 is the treatment year). The denominator is the RMSPE. Taking the reciprocal ensures that countries with higher prediction error have lower values of $\rho_{j}.$ I then used $\rho_{j}$ to calculate a normalized weight,$\pi_{j}$, so that each weight varied from 0 to 1 and all weights sum up to 1, per Equation 2:

Equation 2. $\pi_{j}=\frac{\rho_{j}}{\sum_{j=1}^{J} \rho_{j}}$

I calculate the weighted-mean effect $\tau_{t}$ across all liberalization episodes at a given time point *t* by assigning these weights to each country-level effect $\delta_{jt}$and summing across episodes, per Equation 3:

Equation 3. $\tau_{t}= \sum\pi_{j}.\delta_{jt}$

Appendix 2. Characteristics of liberalizing countries and their comparison units

Out of the 36 trade liberalisation episodes included in my analytic sample, 32 models produced sufficiently good model fit (<√3 average RMSPE) in the pre-treatment period and were included in subsequent analyses. Models for 4 countries – Israel, Chile, Botswana and Nepal – had a pre-treatment prediction error that was above the inclusion threshold and so were excluded from my subsequent analysis and the remainder of the results.

Table 1 below compares the characteristics of countries which liberalized in the study period and their comparison units. Column 1 compares their characteristics with an un-weighted average of the entire donor pool of comparison countries and column 2 compares them with the weighted synthetic control unit (column 2). Table 1 shows that the weighted synthetic control unit more closely resembled treated countries before they liberalised compared with an un-weighted combination of un-treated countries. For example, the mean difference in the pre-treatment mean of GDP per capita across all 32 liberalising countries was 28.6% lower in the synthetic control unit compared with the simple un-weighted mean of comparison countries, and the differences in the pre-treatment mean of the 1, 5 and 10 year lagged child mortality rates were all over 90% lower in the synthetic control unit compared with the un-weighted mean of comparison countries. Table 2 shows this comparison in each country.

Table 1. Mean pre-treatment differences in predictor variables in treated countries and a) an un-weighted combination of comparison countries, and b) the weighted synthetic control unit

| Predictor | Difference in pre-treatment mean | |
| --- | --- | --- |
|  | **Un-weighted controls** | **Synthetic control** |
| GDP per capita | 1,475.37 | 1,052.89 |
| Proportion democracies | 0.37 | 0.26 |
| Rate of urbanization | 0.34 | 0.20 |
| Females with primary education (%) | 0.16 | 0.11 |
| Population growth rate | 0.08 | 0.08 |
| Proportion in conflict | 0.19 | 0.19 |
| Child mortality rate, 1 year before reform | 18.68 | 1.53 |
| Child mortality rate, 5 years before reform | 18.64 | 1.66 |
| Child mortality rate, 10 years before reform | 18.64 | 1.51 |

*Notes*: See Table 1 for measurement and data source for each variable. See Appendix 2 for comparison of predictor means in each country.

Table 2. Comparison of pre-treatment predictor means in treated countries and synthetic controls (T= treatment year)

| ****Country**** | Variable | Treated Country | Sample  Mean | Treated –  Sample Mean | Synthetic Control | Treated – Synthetic Control |
| --- | --- | --- | --- | --- | --- | --- |
| **Albania** | GDP per capita | 1,188.74 | 1,953.50 | 764.76 | 1,322.94 | 134.20 |
|  | Democracy | 0.27 | 0.50 | 0.23 | 0.21 | 0.06 |
|  | Lower-primary education | 22.65 | 39.54 | 16.88 | 41.73 | 19.08 |
|  | Urbanisation | 42.19 | 61.62 | 19.43 | 50.09 | 7.90 |
|  | Population growth | 1.59 | 2.02 | 0.43 | 1.60 | 0.00 |
|  | Conflict | 0.00 | 0.12 | 0.12 | 0.00 | 0.00 |
|  | Mortality, T - 10 | 65.71 | 54.62 | 11.10 | 65.75 | 0.03 |
|  | Mortality, T - 5 | 64.38 | 51.70 | 12.68 | 64.39 | 0.01 |
|  | Mortality, T - 1 | 63.30 | 49.48 | 13.82 | 63.23 | 0.07 |
| **Cameroon** | GDP per capita | 863.31 | 1,968.99 | 1,105.68 | 861.40 | 1.92 |
|  | Democracy | 0.00 | 0.56 | 0.56 | 0.25 | 0.25 |
|  | Lower-primary education | 37.10 | 38.83 | 1.73 | 37.06 | 0.04 |
|  | Urbanisation | 89.61 | 58.73 | 30.88 | 79.18 | 10.43 |
|  | Population growth | 2.99 | 1.95 | 1.03 | 2.41 | 0.58 |
|  | Conflict | 0.00 | 0.11 | 0.11 | 0.01 | 0.01 |
|  | Mortality, T - 10 | 65.52 | 54.06 | 11.46 | 65.52 | 0.00 |
|  | Mortality, T - 5 | 61.49 | 51.18 | 10.31 | 61.49 | 0.00 |
|  | Mortality, T - 1 | 59.18 | 49.17 | 10.02 | 59.19 | 0.00 |
| **Chile** | GDP per capita | 2,072.00 | 466.15 | 1,605.85 | 822.50 | 1,249.50 |
|  | Democracy | 0.64 | 0.30 | 0.34 | 0.00 | 0.64 |
|  | Lower-primary education | 57.22 | 26.16 | 31.06 | 22.24 | 34.98 |
|  | Urbanisation | 66.95 | 112.65 | 45.70 | 140.94 | 73.99 |
|  | Population growth | 1.88 | 2.59 | 0.72 | 2.09 | 0.21 |
|  | Conflict | 0.00 | 0.05 | 0.05 | 0.00 | 0.00 |
|  | Mortality, T - 10 | 27.57 | 78.58 | 51.00 | 61.44 | 33.87 |
|  | Mortality, T - 5 | 24.12 | 76.10 | 51.99 | 59.92 | 35.80 |
|  | Mortality, T - 1 | 21.61 | 74.36 | 52.75 | 58.69 | 37.07 |
| **Colombia** | GDP per capita | 1,462.87 | 1,890.87 | 428.00 | 3,466.61 | 2,003.74 |
|  | Democracy | 1.00 | 0.27 | 0.73 | 0.34 | 0.66 |
|  | Lower-primary education | 54.04 | 40.58 | 13.45 | 49.80 | 4.24 |
|  | Urbanisation | 42.91 | 72.80 | 29.89 | 62.12 | 19.21 |
|  | Population growth | 2.24 | 2.18 | 0.06 | 2.20 | 0.04 |
|  | Conflict | 0.09 | 0.10 | 0.01 | 0.05 | 0.04 |
|  | Mortality, T - 10 | 40.74 | 58.50 | 17.77 | 40.73 | 0.00 |
|  | Mortality, T - 5 | 37.18 | 55.70 | 18.52 | 37.17 | 0.01 |
|  | Mortality, T - 1 | 34.43 | 53.46 | 19.03 | 34.44 | 0.01 |
| **Costa Rica** | GDP per capita | 3,133.35 | 1,841.74 | 1,291.61 | 2,383.53 | 749.82 |
|  | Democracy | 1.00 | 0.27 | 0.73 | 0.82 | 0.18 |
|  | Lower-primary education | 62.31 | 40.34 | 21.97 | 41.59 | 20.72 |
|  | Urbanisation | 21.66 | 73.42 | 51.77 | 63.97 | 42.32 |
|  | Population growth | 2.63 | 2.17 | 0.46 | 2.63 | 0.00 |
|  | Conflict | 0.00 | 0.11 | 0.11 | 0.35 | 0.35 |
|  | Mortality, T - 10 | 58.30 | 57.99 | 0.31 | 58.23 | 0.07 |
|  | Mortality, T - 5 | 56.55 | 55.13 | 1.42 | 56.61 | 0.06 |
|  | Mortality, T - 1 | 54.56 | 52.87 | 1.69 | 54.51 | 0.04 |
| **Ecuador** | GDP per capita | 1,797.33 | 1,932.07 | 134.74 | 1,796.10 | 1.23 |
|  | Democracy | 1.00 | 0.43 | 0.57 | 0.87 | 0.13 |
|  | Lower-primary education | 43.31 | 39.24 | 4.07 | 44.34 | 1.04 |
|  | Urbanisation | 52.94 | 62.91 | 9.98 | 52.63 | 0.30 |
|  | Population growth | 2.47 | 2.01 | 0.45 | 2.42 | 0.04 |
|  | Conflict | 0.00 | 0.13 | 0.13 | 0.05 | 0.05 |
|  | Mortality, T - 10 | 52.11 | 55.57 | 3.45 | 52.09 | 0.02 |
|  | Mortality, T - 5 | 48.01 | 52.79 | 4.78 | 48.02 | 0.01 |
|  | Mortality, T - 1 | 44.91 | 50.51 | 5.60 | 44.90 | 0.01 |
| **El Salvador** | GDP per capita | 1,786.01 | 1,910.48 | 124.47 | 1,785.94 | 0.07 |
|  | Democracy | 0.73 | 0.36 | 0.37 | 0.39 | 0.34 |
|  | Lower-primary education | 48.84 | 39.73 | 9.11 | 48.83 | 0.01 |
|  | Urbanisation | 64.07 | 66.03 | 1.95 | 55.46 | 8.62 |
|  | Population growth | 1.46 | 2.09 | 0.63 | 1.62 | 0.17 |
|  | Conflict | 0.82 | 0.10 | 0.72 | 0.16 | 0.65 |
|  | Mortality, T - 10 | 56.46 | 56.54 | 0.08 | 56.44 | 0.02 |
|  | Mortality, T - 5 | 53.88 | 53.83 | 0.05 | 53.89 | 0.01 |
|  | Mortality, T - 1 | 51.81 | 51.46 | 0.35 | 51.80 | 0.00 |
| **Argentina** | GDP per capita | 2,358.70 | 1,916.03 | 442.67 | 3,593.45 | 1,234.75 |
|  | Democracy | 0.82 | 0.44 | 0.38 | 0.67 | 0.15 |
|  | Lower-primary education | 58.02 | 38.82 | 19.20 | 51.84 | 6.18 |
|  | Urbanisation | 28.42 | 63.61 | 35.20 | 28.34 | 0.08 |
|  | Population growth | 1.51 | 2.04 | 0.53 | 0.97 | 0.54 |
|  | Conflict | 0.00 | 0.13 | 0.13 | 0.01 | 0.01 |
|  | Mortality, T - 10 | 16.69 | 56.58 | 39.89 | 16.66 | 0.03 |
|  | Mortality, T - 5 | 14.56 | 53.74 | 39.19 | 14.59 | 0.03 |
|  | Mortality, T - 1 | 13.02 | 51.42 | 38.40 | 13.01 | 0.00 |
| **Ghana** | GDP per capita | 271.55 | 2,013.89 | 1,742.33 | 1,392.93 | 1,121.38 |
|  | Democracy | 0.18 | 0.29 | 0.11 | 0.12 | 0.07 |
|  | Lower-primary education | 11.73 | 43.30 | 31.58 | 23.96 | 12.23 |
|  | Urbanisation | 102.03 | 69.25 | 32.78 | 101.91 | 0.11 |
|  | Population growth | 2.55 | 2.18 | 0.37 | 2.55 | 0.00 |
|  | Conflict | 0.00 | 0.09 | 0.09 | 0.05 | 0.05 |
|  | Mortality, T - 10 | 69.95 | 56.58 | 13.37 | 70.03 | 0.07 |
|  | Mortality, T - 5 | 68.84 | 53.79 | 15.05 | 68.89 | 0.05 |
|  | Mortality, T - 1 | 67.79 | 51.73 | 16.06 | 67.69 | 0.10 |
| **Guatemala** | GDP per capita | 1,778.54 | 1,894.97 | 116.43 | 1,536.35 | 242.19 |
|  | Democracy | 0.27 | 0.33 | 0.06 | 0.44 | 0.17 |
|  | Lower-primary education | 33.44 | 40.37 | 6.93 | 33.51 | 0.07 |
|  | Urbanisation | 79.08 | 67.44 | 11.64 | 81.37 | 2.28 |
|  | Population growth | 2.57 | 2.10 | 0.47 | 2.30 | 0.27 |
|  | Conflict | 0.00 | 0.13 | 0.13 | 0.30 | 0.30 |
|  | Mortality, T - 10 | 62.87 | 56.94 | 5.94 | 62.97 | 0.10 |
|  | Mortality, T - 5 | 61.62 | 54.17 | 7.45 | 61.60 | 0.03 |
|  | Mortality, T - 1 | 60.07 | 51.83 | 8.24 | 60.06 | 0.01 |
| **Guyana** | GDP per capita | 763.14 | 1,923.98 | 1,160.84 | 773.73 | 10.59 |
|  | Democracy | 0.00 | 0.34 | 0.34 | 0.00 | 0.00 |
|  | Lower-primary education | 64.80 | 39.48 | 25.32 | 40.76 | 24.04 |
|  | Urbanisation | 51.64 | 68.23 | 16.59 | 80.19 | 28.55 |
|  | Population growth | -0.34 | 2.18 | 2.52 | 3.29 | 3.63 |
|  | Conflict | 0.00 | 0.13 | 0.13 | 0.00 | 0.00 |
|  | Mortality, T - 10 | 69.71 | 56.74 | 12.96 | 71.18 | 1.47 |
|  | Mortality, T - 5 | 69.79 | 53.94 | 15.85 | 69.67 | 0.12 |
|  | Mortality, T - 1 | 70.15 | 51.54 | 18.61 | 69.52 | 0.62 |
| **Honduras** | GDP per capita | 932.33 | 1,956.78 | 1,024.45 | 1,117.17 | 184.85 |
|  | Democracy | 1.00 | 0.43 | 0.57 | 0.36 | 0.64 |
|  | Lower-primary education | 52.97 | 38.96 | 14.01 | 34.34 | 18.63 |
|  | Urbanisation | 53.98 | 62.88 | 8.90 | 76.08 | 22.10 |
|  | Population growth | 2.97 | 2.00 | 0.97 | 2.13 | 0.84 |
|  | Conflict | 0.00 | 0.13 | 0.13 | 0.01 | 0.01 |
|  | Mortality, T - 10 | 64.57 | 55.21 | 9.36 | 64.56 | 0.00 |
|  | Mortality, T - 5 | 61.68 | 52.40 | 9.29 | 61.68 | 0.00 |
|  | Mortality, T - 1 | 59.54 | 50.09 | 9.45 | 59.54 | 0.00 |
| **Hungary** | GDP per capita | 5,319.15 | 1,822.84 | 3,496.31 | 1,976.00 | 3,343.15 |
|  | Democracy | 0.18 | 0.41 | 0.23 | 0.30 | 0.12 |
|  | Lower-primary education | 65.48 | 38.90 | 26.58 | 56.23 | 9.25 |
|  | Urbanisation | 20.60 | 65.51 | 44.91 | 34.59 | 13.99 |
|  | Population growth | -0.29 | 2.11 | 2.40 | 0.27 | 0.55 |
|  | Conflict | 0.00 | 0.13 | 0.13 | 0.00 | 0.00 |
|  | Mortality, T - 10 | 35.81 | 56.57 | 20.76 | 35.84 | 0.03 |
|  | Mortality, T - 5 | 34.91 | 53.77 | 18.87 | 34.89 | 0.02 |
|  | Mortality, T - 1 | 34.19 | 51.39 | 17.20 | 34.20 | 0.01 |
| **Israel** | GDP per capita | 11,337.90 | 1,668.06 | 9,669.84 | 2,618.32 | 8,719.59 |
|  | Democracy | 1.00 | 0.27 | 0.73 | 0.09 | 0.91 |
|  | Lower-primary education | 27.95 | 42.80 | 14.84 | 58.11 | 30.15 |
|  | Urbanisation | 16.08 | 71.94 | 55.85 | 36.67 | 20.59 |
|  | Population growth | 2.05 | 2.20 | 0.14 | 0.59 | 1.47 |
|  | Conflict | 0.09 | 0.09 | 0.01 | 0.00 | 0.09 |
|  | Mortality, T - 10 | 13.36 | 58.35 | 44.99 | 16.61 | 3.26 |
|  | Mortality, T - 5 | 11.42 | 55.58 | 44.16 | 14.61 | 3.19 |
|  | Mortality, T - 1 | 10.28 | 53.53 | 43.25 | 13.15 | 2.87 |
| **Jamaica** | GDP per capita | 2,779.54 | 1,882.10 | 897.44 | 2,779.14 | 0.40 |
|  | Democracy | 1.00 | 0.35 | 0.65 | 0.35 | 0.65 |
|  | Lower-primary education | 62.55 | 39.34 | 23.22 | 38.80 | 23.75 |
|  | Urbanisation | 29.82 | 67.00 | 37.19 | 71.65 | 41.83 |
|  | Population growth | 1.15 | 2.10 | 0.95 | 2.77 | 1.61 |
|  | Conflict | 0.00 | 0.12 | 0.12 | 0.02 | 0.02 |
|  | Mortality, T - 10 | 53.81 | 56.61 | 2.80 | 53.74 | 0.07 |
|  | Mortality, T - 5 | 51.78 | 53.89 | 2.11 | 51.84 | 0.06 |
|  | Mortality, T - 1 | 50.96 | 51.48 | 0.52 | 50.92 | 0.04 |
| **Kenya** | GDP per capita | 419.10 | 1,981.68 | 1,562.58 | 361.19 | 57.91 |
|  | Democracy | 0.00 | 0.56 | 0.56 | 0.29 | 0.29 |
|  | Lower-primary education | 44.81 | 38.61 | 6.20 | 24.92 | 19.89 |
|  | Urbanisation | 65.10 | 59.43 | 5.67 | 84.08 | 18.98 |
|  | Population growth | 3.51 | 1.94 | 1.57 | 1.22 | 2.29 |
|  | Conflict | 0.00 | 0.11 | 0.11 | 0.00 | 0.00 |
|  | Mortality, T - 10 | 84.12 | 53.53 | 30.59 | 84.11 | 0.01 |
|  | Mortality, T - 5 | 83.62 | 50.55 | 33.07 | 83.33 | 0.28 |
|  | Mortality, T - 1 | 82.66 | 48.50 | 34.16 | 82.55 | 0.11 |
| **Mali** | GDP per capita | 220.90 | 1,939.47 | 1,718.57 | 602.81 | 381.91 |
|  | Democracy | 0.00 | 0.34 | 0.34 | 0.09 | 0.09 |
|  | Lower-primary education | 7.59 | 41.11 | 33.52 | 16.12 | 8.52 |
|  | Urbanisation | 151.05 | 65.39 | 85.66 | 116.47 | 34.58 |
|  | Population growth | 1.81 | 2.12 | 0.31 | 2.15 | 0.34 |
|  | Conflict | 0.00 | 0.13 | 0.13 | 0.16 | 0.16 |
|  | Mortality, T - 10 | 82.46 | 56.38 | 26.08 | 82.42 | 0.04 |
|  | Mortality, T - 5 | 80.03 | 53.65 | 26.38 | 80.05 | 0.02 |
|  | Mortality, T - 1 | 77.93 | 51.32 | 26.61 | 77.92 | 0.01 |
| **Mauritania** | GDP per capita | 418.42 | 2,016.20 | 1,597.78 | 1,254.11 | 835.69 |
|  | Democracy | 0.00 | 0.66 | 0.66 | 0.21 | 0.21 |
|  | Lower-primary education | 32.16 | 38.29 | 6.12 | 36.29 | 4.12 |
|  | Urbanisation | 79.34 | 56.10 | 23.24 | 77.71 | 1.63 |
|  | Population growth | 2.78 | 1.92 | 0.87 | 2.89 | 0.11 |
|  | Conflict | 0.00 | 0.08 | 0.08 | 0.00 | 0.00 |
|  | Mortality, T - 10 | 65.01 | 52.91 | 12.09 | 64.59 | 0.41 |
|  | Mortality, T - 5 | 58.68 | 50.12 | 8.56 | 58.80 | 0.12 |
|  | Mortality, T - 1 | 55.54 | 48.42 | 7.12 | 55.71 | 0.17 |
| **Mexico** | GDP per capita | 5,186.96 | 1,781.34 | 3,405.62 | 1,873.68 | 3,313.28 |
|  | Democracy | 0.00 | 0.30 | 0.30 | 0.01 | 0.01 |
|  | Lower-primary education | 47.69 | 40.77 | 6.92 | 47.65 | 0.04 |
|  | Urbanisation | 53.97 | 72.47 | 18.50 | 50.50 | 3.47 |
|  | Population growth | 2.36 | 2.18 | 0.18 | 1.86 | 0.50 |
|  | Conflict | 0.00 | 0.11 | 0.11 | 0.00 | 0.00 |
|  | Mortality, T - 10 | 36.51 | 58.63 | 22.12 | 36.03 | 0.48 |
|  | Mortality, T - 5 | 33.13 | 55.82 | 22.69 | 32.88 | 0.26 |
|  | Mortality, T - 1 | 31.05 | 53.56 | 22.51 | 31.35 | 0.30 |
| **Mozambique** | GDP per capita | 138.96 | 2,024.19 | 1,885.23 | 257.18 | 118.22 |
|  | Democracy | 0.18 | 0.65 | 0.47 | 0.38 | 0.19 |
|  | Lower-primary education | 13.67 | 38.81 | 25.14 | 10.97 | 2.71 |
|  | Urbanisation | 158.41 | 53.84 | 104.57 | 130.38 | 28.03 |
|  | Population growth | 1.86 | 1.94 | 0.08 | 2.04 | 0.18 |
|  | Conflict | 0.64 | 0.06 | 0.57 | 0.01 | 0.63 |
|  | Mortality, T - 10 | 81.67 | 52.44 | 29.24 | 78.48 | 3.20 |
|  | Mortality, T - 5 | 75.00 | 49.65 | 25.35 | 76.16 | 1.16 |
|  | Mortality, T - 1 | 73.02 | 47.92 | 25.10 | 74.38 | 1.37 |
| **Nepal** | GDP per capita | 160.75 | 1,978.83 | 1,818.08 | 243.24 | 82.49 |
|  | Democracy | 0.18 | 0.46 | 0.27 | 0.09 | 0.09 |
|  | Lower-primary education | 3.12 | 40.39 | 37.26 | 4.87 | 1.75 |
|  | Urbanisation | 115.12 | 61.14 | 53.98 | 138.31 | 23.19 |
|  | Population growth | 2.33 | 2.02 | 0.31 | 2.86 | 0.53 |
|  | Conflict | 0.00 | 0.13 | 0.13 | 0.00 | 0.00 |
|  | Mortality, T - 10 | 93.62 | 54.38 | 39.24 | 86.34 | 7.28 |
|  | Mortality, T - 5 | 92.34 | 51.52 | 40.82 | 85.24 | 7.10 |
|  | Mortality, T - 1 | 91.15 | 49.19 | 41.96 | 84.63 | 6.51 |
| **Nicaragua** | GDP per capita | 950.74 | 1,956.25 | 1,005.51 | 1,344.35 | 393.60 |
|  | Democracy | 0.18 | 0.46 | 0.27 | 0.18 | 0.00 |
|  | Lower-primary education | 30.75 | 39.60 | 8.85 | 31.54 | 0.80 |
|  | Urbanisation | 59.72 | 62.72 | 3.00 | 60.14 | 0.42 |
|  | Population growth | 2.41 | 2.01 | 0.40 | 2.17 | 0.24 |
|  | Conflict | 0.55 | 0.12 | 0.43 | 0.01 | 0.54 |
|  | Mortality, T - 10 | 49.82 | 55.63 | 5.81 | 49.85 | 0.03 |
|  | Mortality, T - 5 | 48.62 | 52.77 | 4.15 | 48.62 | 0.00 |
|  | Mortality, T - 1 | 47.66 | 50.43 | 2.77 | 47.65 | 0.01 |
| **Niger** | GDP per capita | 218.22 | 2,000.85 | 1,782.63 | 330.10 | 111.88 |
|  | Democracy | 0.36 | 0.60 | 0.23 | 0.37 | 0.00 |
|  | Lower-primary education | 5.91 | 39.43 | 33.52 | 22.57 | 16.66 |
|  | Urbanisation | 136.45 | 55.91 | 80.54 | 84.07 | 52.38 |
|  | Population growth | 3.04 | 1.93 | 1.11 | 1.69 | 1.34 |
|  | Conflict | 0.00 | 0.09 | 0.09 | 0.00 | 0.00 |
|  | Mortality, T - 10 | 85.69 | 52.92 | 32.77 | 85.78 | 0.09 |
|  | Mortality, T - 5 | 84.71 | 49.94 | 34.77 | 84.93 | 0.22 |
|  | Mortality, T - 1 | 84.39 | 48.02 | 36.37 | 83.98 | 0.41 |
| **Paraguay** | GDP per capita | 942.76 | 1,934.58 | 991.82 | 1,971.35 | 1,028.59 |
|  | Democracy | 0.09 | 0.37 | 0.28 | 0.27 | 0.18 |
|  | Lower-primary education | 63.39 | 39.31 | 24.08 | 43.38 | 20.01 |
|  | Urbanisation | 44.44 | 66.59 | 22.15 | 46.48 | 2.04 |
|  | Population growth | 2.81 | 2.06 | 0.76 | 1.92 | 0.90 |
|  | Conflict | 0.00 | 0.12 | 0.12 | 0.01 | 0.01 |
|  | Mortality, T - 10 | 58.86 | 56.47 | 2.39 | 58.85 | 0.01 |
|  | Mortality, T - 5 | 55.75 | 53.78 | 1.98 | 55.76 | 0.01 |
|  | Mortality, T - 1 | 52.79 | 51.43 | 1.36 | 52.79 | 0.00 |
| **Peru** | GDP per capita | 2,227.36 | 1,919.78 | 307.58 | 5,738.97 | 3,511.61 |
|  | Democracy | 1.00 | 0.43 | 0.57 | 0.62 | 0.38 |
|  | Lower-primary education | 36.52 | 39.43 | 2.92 | 36.49 | 0.02 |
|  | Urbanisation | 67.54 | 62.50 | 5.04 | 61.60 | 5.94 |
|  | Population growth | 2.27 | 2.02 | 0.25 | 2.11 | 0.16 |
|  | Conflict | 0.64 | 0.11 | 0.52 | 0.18 | 0.46 |
|  | Mortality, T - 10 | 34.82 | 56.06 | 21.24 | 35.28 | 0.46 |
|  | Mortality, T - 5 | 32.72 | 53.22 | 20.50 | 32.84 | 0.12 |
|  | Mortality, T - 1 | 31.10 | 50.90 | 19.80 | 30.96 | 0.14 |
| **Philippines** | GDP per capita | 901.25 | 1,920.03 | 1,018.78 | 1,718.48 | 817.23 |
|  | Democracy | 0.27 | 0.33 | 0.06 | 0.66 | 0.38 |
|  | Lower-primary education | 49.20 | 39.92 | 9.27 | 45.79 | 3.41 |
|  | Urbanisation | 50.76 | 68.25 | 17.50 | 64.80 | 14.04 |
|  | Population growth | 2.72 | 2.09 | 0.62 | 2.18 | 0.54 |
|  | Conflict | 0.73 | 0.10 | 0.62 | 0.41 | 0.32 |
|  | Mortality, T - 10 | 63.37 | 56.92 | 6.44 | 63.32 | 0.05 |
|  | Mortality, T - 5 | 59.21 | 54.24 | 4.97 | 59.23 | 0.02 |
|  | Mortality, T - 1 | 54.67 | 51.98 | 2.69 | 54.67 | 0.01 |
| **Benin** | GDP per capita | 367.64 | 1,964.31 | 1,596.67 | 1,323.19 | 955.55 |
|  | Democracy | 0.00 | 0.42 | 0.42 | 0.02 | 0.02 |
|  | Lower-primary education | 8.12 | 40.54 | 32.42 | 21.51 | 13.40 |
|  | Urbanisation | 118.66 | 62.71 | 55.96 | 118.63 | 0.03 |
|  | Population growth | 2.94 | 2.02 | 0.92 | 1.86 | 1.08 |
|  | Conflict | 0.00 | 0.13 | 0.13 | 0.27 | 0.27 |
|  | Mortality, T - 10 | 72.66 | 55.52 | 17.15 | 72.64 | 0.02 |
|  | Mortality, T - 5 | 69.20 | 52.79 | 16.41 | 69.22 | 0.02 |
|  | Mortality, T - 1 | 66.27 | 50.47 | 15.80 | 66.26 | 0.01 |
| **Poland** | GDP per capita | 3,596.38 | 1,872.06 | 1,724.32 | 3,596.08 | 0.31 |
|  | Democracy | 0.18 | 0.41 | 0.23 | 0.21 | 0.03 |
|  | Lower-primary education | 53.72 | 39.23 | 14.48 | 53.72 | 0.00 |
|  | Urbanisation | 17.98 | 65.58 | 47.60 | 37.79 | 19.81 |
|  | Population growth | 0.71 | 2.08 | 1.37 | 0.71 | 0.00 |
|  | Conflict | 0.00 | 0.13 | 0.13 | 0.00 | 0.00 |
|  | Mortality, T - 10 | 41.91 | 56.40 | 14.48 | 41.91 | 0.01 |
|  | Mortality, T - 5 | 40.06 | 53.63 | 13.57 | 40.10 | 0.04 |
|  | Mortality, T - 1 | 38.78 | 51.26 | 12.48 | 38.74 | 0.03 |
| **South Africa** | GDP per capita | 2,530.39 | 1,911.12 | 619.26 | 2,530.62 | 0.24 |
|  | Democracy | 1.00 | 0.43 | 0.57 | 0.56 | 0.44 |
|  | Lower-primary education | 47.02 | 39.13 | 7.89 | 47.03 | 0.01 |
|  | Urbanisation | 54.20 | 62.88 | 8.68 | 47.88 | 6.32 |
|  | Population growth | 2.36 | 2.02 | 0.34 | 2.36 | 0.00 |
|  | Conflict | 0.55 | 0.12 | 0.43 | 0.01 | 0.53 |
|  | Mortality, T - 10 | 51.41 | 55.59 | 4.18 | 51.60 | 0.19 |
|  | Mortality, T - 5 | 50.10 | 52.73 | 2.63 | 49.97 | 0.12 |
|  | Mortality, T - 1 | 47.96 | 50.42 | 2.46 | 48.05 | 0.09 |
| **Bolivia** | GDP per capita | 987.41 | 1,991.52 | 1,004.11 | 1,480.87 | 493.46 |
|  | Democracy | 0.36 | 0.29 | 0.08 | 0.05 | 0.31 |
|  | Lower-primary education | 27.54 | 42.81 | 15.27 | 36.11 | 8.57 |
|  | Urbanisation | 112.95 | 68.91 | 44.04 | 111.55 | 1.39 |
|  | Population growth | 2.15 | 2.19 | 0.04 | 2.15 | 0.00 |
|  | Conflict | 0.00 | 0.09 | 0.09 | 0.00 | 0.00 |
|  | Mortality, T - 10 | 58.69 | 56.93 | 1.76 | 58.11 | 0.58 |
|  | Mortality, T - 5 | 54.55 | 54.23 | 0.32 | 54.41 | 0.14 |
|  | Mortality, T - 1 | 50.50 | 52.28 | 1.78 | 51.53 | 1.04 |
| **Uganda** | GDP per capita | 169.53 | 1,891.73 | 1,722.21 | 236.76 | 67.23 |
|  | Democracy | 0.46 | 0.33 | 0.12 | 0.04 | 0.42 |
|  | Lower-primary education | 33.28 | 40.18 | 6.90 | 5.63 | 27.65 |
|  | Urbanisation | 121.42 | 67.77 | 53.65 | 121.54 | 0.12 |
|  | Population growth | 3.15 | 2.11 | 1.04 | 2.37 | 0.79 |
|  | Conflict | 0.73 | 0.12 | 0.61 | 0.00 | 0.72 |
|  | Mortality, T - 10 | 92.72 | 57.10 | 35.62 | 92.98 | 0.26 |
|  | Mortality, T - 5 | 91.53 | 54.38 | 37.15 | 91.48 | 0.05 |
|  | Mortality, T - 1 | 90.12 | 52.06 | 38.06 | 90.13 | 0.01 |
| **Uruguay** | GDP per capita | 2,784.92 | 1,895.24 | 889.68 | 7,004.14 | 4,219.22 |
|  | Democracy | 0.55 | 0.40 | 0.15 | 0.85 | 0.31 |
|  | Lower-primary education | 54.04 | 39.22 | 14.82 | 43.68 | 10.36 |
|  | Urbanisation | 26.11 | 65.35 | 39.24 | 21.63 | 4.48 |
|  | Population growth | 0.65 | 2.08 | 1.44 | 1.69 | 1.04 |
|  | Conflict | 0.00 | 0.13 | 0.13 | 0.04 | 0.04 |
|  | Mortality, T - 10 | 14.61 | 57.18 | 42.57 | 14.49 | 0.12 |
|  | Mortality, T - 5 | 12.81 | 54.40 | 41.60 | 12.75 | 0.05 |
|  | Mortality, T - 1 | 11.37 | 52.04 | 40.67 | 11.71 | 0.34 |
| **Zambia** | GDP per capita | 489.73 | 1,979.67 | 1,489.93 | 2,605.18 | 2,115.45 |
|  | Democracy | 0.27 | 0.55 | 0.28 | 0.31 | 0.04 |
|  | Lower-primary education | 39.55 | 38.76 | 0.79 | 53.47 | 13.93 |
|  | Urbanisation | 109.34 | 58.17 | 51.17 | 42.40 | 66.93 |
|  | Population growth | 2.96 | 1.95 | 1.00 | -0.28 | 3.24 |
|  | Conflict | 0.00 | 0.11 | 0.11 | 0.00 | 0.00 |
|  | Mortality, T - 10 | 60.25 | 54.21 | 6.03 | 60.48 | 0.24 |
|  | Mortality, T - 5 | 60.49 | 51.21 | 9.28 | 60.77 | 0.27 |
|  | Mortality, T - 1 | 61.49 | 49.10 | 12.39 | 61.00 | 0.49 |
| **Botswana** | GDP per capita | 602.73 | 2,261.02 | 1,658.29 | 242.47 | 360.27 |
|  | Democracy | 1.00 | 0.43 | 0.57 | 0.00 | 1.00 |
|  | Lower-primary education | 42.12 | 40.17 | 1.96 | 4.16 | 37.97 |
|  | Urbanisation | 72.74 | 82.17 | 9.43 | 183.21 | 110.47 |
|  | Population growth | 3.53 | 2.18 | 1.35 | 1.68 | 1.85 |
|  | Conflict | 0.00 | 0.04 | 0.04 | 0.00 | 0.00 |
|  | Mortality, T - 10 | 93.19 | 58.19 | 35.00 | 86.03 | 7.16 |
|  | Mortality, T - 5 | 88.89 | 56.00 | 32.89 | 84.16 | 4.74 |
|  | Mortality, T - 1 | 85.51 | 54.33 | 31.19 | 82.46 | 3.05 |
| **Brazil** | GDP per capita | 2,638.48 | 1,908.03 | 730.45 | 2,676.29 | 37.81 |
|  | Democracy | 0.64 | 0.44 | 0.19 | 0.64 | 0.00 |
|  | Lower-primary education | 58.14 | 38.81 | 19.33 | 49.68 | 8.47 |
|  | Urbanisation | 59.79 | 62.72 | 2.93 | 38.73 | 21.07 |
|  | Population growth | 2.04 | 2.02 | 0.02 | 1.98 | 0.06 |
|  | Conflict | 0.00 | 0.13 | 0.13 | 0.00 | 0.00 |
|  | Mortality, T - 10 | 33.63 | 56.09 | 22.46 | 33.49 | 0.14 |
|  | Mortality, T - 5 | 29.30 | 53.32 | 24.02 | 29.51 | 0.21 |
|  | Mortality, T - 1 | 26.08 | 51.05 | 24.97 | 26.22 | 0.14 |
| **Bulgaria** | GDP per capita | 2,490.96 | 1,912.25 | 578.72 | 2,486.31 | 4.65 |
|  | Democracy | 0.18 | 0.46 | 0.27 | 0.18 | 0.00 |
|  | Lower-primary education | 46.22 | 39.16 | 7.06 | 42.77 | 3.44 |
|  | Urbanisation | 19.81 | 63.86 | 44.05 | 47.18 | 27.37 |
|  | Population growth | -0.24 | 2.09 | 2.33 | 2.10 | 2.34 |
|  | Conflict | 0.00 | 0.13 | 0.13 | 0.01 | 0.01 |
|  | Mortality, T - 10 | 37.40 | 55.99 | 18.59 | 37.37 | 0.03 |
|  | Mortality, T - 5 | 35.00 | 53.16 | 18.16 | 35.03 | 0.03 |
|  | Mortality, T - 1 | 33.62 | 50.83 | 17.21 | 33.59 | 0.03 |

Appendix 3. Countries and year of liberalization

| Country | Year |  | Country | Year |
| --- | --- | --- | --- | --- |
| Albania | 1992 |  | Mexico | 1986 |
| Cameroon | 1993 |  | Mozambique | 1995 |
| Chile | 1976 |  | Nepal | 1991 |
| Colombia | 1986 |  | Nicaragua | 1991 |
| Costa Rica | 1986 |  | Niger | 1994 |
| Ecuador | 1991 |  | Paraguay | 1989 |
| El Salvador | 1989 |  | Peru | 1991 |
| Argentina | 1991 |  | Philippines | 1988 |
| Ghana | 1985 |  | Benin | 1990 |
| Guatemala | 1988 |  | Poland | 1990 |
| Guyana | 1988 |  | South Africa | 1991 |
| Honduras | 1991 |  | Bolivia | 1985 |
| Hungary | 1990 |  | Uganda | 1988 |
| Israel | 1985 |  | Uruguay | 1990 |
| Jamaica | 1989 |  | Zambia | 1993 |
| Kenya | 1993 |  | Botswana | 1979 |
| Mali | 1988 |  | Brazil | 1991 |
| Mauritania | 1995 |  | Bulgaria | 1991 |

Appendix 4. Alternative RMSPE cut-offs

| ****Test**** | ****Average effect of trade liberalization, %**** | ****95% confidence interval**** |
| --- | --- | --- |
| RMSPE cut-off:  mean pre-intervention RMSPE | -0.15 | -3.93 to 1.47 |
| RMSPE cut-off:  3 *x* mean pre-intervention RMSPE | -0.16 | -4.21 to 1.58 |
| Equal country weights | -0.56 | -2.04 to 2.18 |

Appendix 5. Un-weighted treatment effects and 95% confidence intervals

*Notes:* 95% confidence intervals are estimated from the (un-weighted) mean effect in 5,000 placebo experiments.

Appendix 6. Leave-one-out analysis of liberalisation effects by country in all donor-pool specifications

*Notes:* The RMSPE Ratio is the ratio of the pre-intervention RMSPE in the model using the specified comparison group (triangles=all countries, circles=same region) to the RMSPE in my original specification using all countries. Higher ratios indicate better model fit.

Appendix 7. Leave-one-out analysis of liberalisation effects by country: weighted mean across all alternative donor-pool specifications

*Notes:* The RMSPE Ratio is the ratio of the pre-intervention RMSPE in the model using the specified comparison group (triangles=all countries, circles=same region) to the RMSPE in my original specification using all countries. Higher ratios indicate better model fit.
